# Do Gastric Balloons Have a Future? Intragastric Balloons in the Era of Highly Effective Obesity Pharmacotherapy

**DOI:** 10.3390/medicina62101916

**Published:** 2026-10-04

**Authors:** Rhea Sibal, G. Balamurugan, Mohammed Aradaib, Yitka Graham, Kamal Mahawar

**Affiliations:** 1 Faculty of Life Sciences and Medicine, King’s College London, London SE1 1UL, UK; 2 Department of General Surgery, South Tyneside and Sunderland NHS Foundation Trust, Sunderland SR4 7TP, UK; surgeondrbala@gmail.com (G.B.); mohammedaradaib@gmail.com (M.A.);; 3 Helen McArdle Nursing and Care Research Institute, University of Sunderland, Sunderland SR6 0DD, UK; 4 Faculty of Biomedical Sciences, Austral University, Buenos Aires B1406, Argentina; 5 Faculty of Psychology, University of Anahuac, Mexico City 52786, Mexico

**Keywords:** endoscopic bariatric therapy, incretin therapy, intragastric balloon, pharmacotherapy, metabolic surgery, weight loss, obesity

## Abstract

Intragastric balloons (IGBs) were developed to bridge the gap between lifestyle intervention and Metabolic and Bariatric Surgery (MBS), but the advent of highly efficacious pharmacotherapies and Endoscopic Sleeve Gastroplasty (ESG) has challenged their role. This narrative review synthesises contemporary evidence and guideline recommendations regarding IGBs, focusing on short-term efficacy, durability, safety, psychological aspects, and health system considerations in comparison with Glucagon-Like Peptide-1 Receptor Agonists (GLP-1 RAs), dual GIP (Glucose-dependent Insulinotropic Polypeptide)/GLP-1 RAs, ESG, and MBS. Across randomised trials and meta-analyses, IGBs consistently produce modest short-term weight loss of approximately 10–15% total body weight at six months, with frequent weight regain following removal and limited long-term benefit. In contrast, modern pharmacotherapy achieves greater and more durable weight loss with fewer procedural risks. Current guidelines therefore position IGBs as adjunctive or bridging interventions reserved for selected patient subgroups, rather than as a dominant intermediate therapy. Overall, current evidence supports a selective role for IGBs as bridging, adjunctive, or combination interventions within multimodal obesity pathways, particularly for patients who cannot access or tolerate pharmacotherapy or who require a behavioural and metabolic bridge to surgery.

## 1. Introduction

Obesity is a chronic, relapsing disease associated with significant morbidity and mortality [1]. Historically, treatment options have centred on lifestyle modification and, more recently, pharmacotherapy and metabolic and bariatric surgery (MBS), yet a substantial treatment gap has persisted. Endoscopic bariatric therapies (EBTs), particularly intragastric balloons (IGBs), were introduced to address this unmet need, reflecting growing interest in treatments that are both effective and less invasive while avoiding surgical risks [2]. IGBs emerged as one such option, offering a reversible, endoscopic approach to weight management. Although most IGB systems require endoscopy for placement and removal, self-dissolving swallowable balloons that can be positioned under radiological guidance are also available [3].

The mechanism of action of IGBs is multifactorial. These devices are placed within the gastric lumen and filled with fluid or gas, occupying space within the stomach and promoting early satiety. In addition to gastric volume occupation, IGBs delay gastric emptying and enhance the sensation of fullness, thereby reducing overall food consumption and, consequently, caloric intake [4]. There is also evidence that balloon placement may influence gastrointestinal hormone signalling, including pathways involving ghrelin, leptin, cholecystokinin, peptide YY, pancreatic polypeptide, and glucagon-like peptide-1 (GLP-1), with combined mechanical and neurohormonal effects contributing to appetite suppression and weight reduction [5,6].

IGBs are typically indicated in individuals with obesity, particularly those with a body mass index (BMI) ≥30 kg/m^2^ who are at increased risk of obesity-related comorbidities, or in those who have not achieved adequate weight loss through lifestyle intervention alone [7]. Recent guidance from the American Society for Gastrointestinal Endoscopy (ASGE) and European Society of Gastrointestinal Endoscopy (ESGE) positions endoscopic therapies, including IGBs, as adjuncts rather than definitive treatments [8], reinforcing their role as selective rather than primary interventions.

The therapeutic landscape has shifted dramatically towards glucagon-like peptide-1 receptor agonists (GLP-1 RAs), with a paradigm change driven by highly efficacious incretin-based therapies, particularly GLP-1 RAs and dual GIP (glucose-dependent insulinotropic polypeptide)/GLP-1 RAs. In keeping with the 2022 American Society for Metabolic and Bariatric Surgery (ASMBS)–International Federation for the Surgery of Obesity and Metabolic Disorders (IFSO) guidelines, which emphasise a chronic disease model requiring long-term therapy, with increasing recognition of pharmacotherapy as a central pillar [9]. Semaglutide 2.4 mg (GLP-1 RA) once weekly has demonstrated substantial and durable weight reduction in the landmark STEP-1 trial—a randomised, double-blind, placebo-controlled study enrolling 1961 adults with overweight or obesity—in which participants achieved a mean weight loss of 14.9% at 68 weeks, with 86.4% achieving ≥5% and 69.1% achieving ≥10% total body weight loss [10]. Critically, the STEP-5 trial demonstrated that these reductions were sustained over 104 weeks, with a mean weight loss of 15.2% maintained at two years, underscoring the durability of continued therapy [11]. More recently, tirzepatide—a novel dual GIP and GLP-1 RA—has demonstrated superior efficacy in the SURMOUNT-1 trial, a phase 3 randomised controlled trial of 2539 adults with obesity, achieving mean weight reductions of up to 20.9% at 72 weeks with the 15 mg dose, with 91% of participants achieving ≥5% weight loss [12].

Within this evolving framework, the role of IGBs must now be considered in the context of a rapidly changing therapeutic landscape. Accordingly, this review aims to critically evaluate the role of IGBs within the modern obesity treatment paradigm by directly comparing their efficacy, safety, and durability with contemporary pharmacological and endoscopic interventions. Emphasis is placed on the magnitude and sustainability of weight loss, impact on metabolic comorbidities, and patient tolerability, as well as practical considerations such as accessibility and cost.

## 2. Literature Search and Selection

A narrative review approach was chosen to provide a meaningful synthesis of published studies on IGBs within a rapidly evolving therapeutic landscape. The literature search and selection were undertaken in three broad stages: identifying relevant publications, screening for eligibility, and synthesising findings across endoscopic, pharmacological, and surgical obesity treatments.

A comprehensive search was performed in March 2026 using PubMed, supplemented by targeted hand-searching of reference lists from key reviews, meta-analyses, and international guideline documents. Only English-language publications were considered. Search terms focused on IGBs and obesity treatment, including device names (“intragastric balloon”, “gastric balloon”, “Orbera”, “Obalon”, “Elipse”), obesity and treatment terms (“obesity”, “overweight”, “anti-obesity agent”, “semaglutide”, “liraglutide”, “tirzepatide”, “endoscopic sleeve gastroplasty”, “bariatric surgery”), and key outcome concepts such as “weight loss”, “durability”, “weight regain”, “safety”, and “adverse events”.

Studies were excluded if they lacked clinical outcome data, were animal-only, were non-English, or did not report on IGBs or relevant comparator therapies. Eligible sources encompassed randomised controlled trials, observational studies, systematic reviews, meta-analyses, and practice guidelines. Guidance documents from professional bodies (ASMBS, IFSO, ASGE, ESGE, and the National Institute for Health and Care Excellence [NICE]) were included to provide a contemporary clinical framework. From each publication, data were extracted on weight loss outcomes, durability following treatment, adverse-event profiles, and guideline-based recommendations, which were then integrated narratively in the subsequent sections of this review.

## 3. Evolution and Current Positioning of IGBs

IGBs have undergone significant technological refinement since their initial introduction. Early devices were associated with high complication rates, including spontaneous deflation and intestinal obstruction, which limited their clinical acceptance [13]. Contemporary systems, including fluid-filled balloons such as Orbera, gas-filled systems such as Obalon, and swallowable devices such as the Allurion balloon (formerly Elipse^®^, Allurion Technologies), have demonstrated improved safety profiles and procedural simplicity, facilitating wider adoption, particularly in outpatient settings [14].

In parallel with these device-level advances, recent international guidance has clarified the clinical context for IGB use. The ASGE–ESGE guideline on primary endoscopic bariatric and metabolic therapies suggests the use of IGBs, in conjunction with lifestyle modification, for adults with a BMI ≥ 30 kg/m^2^ or with a BMI of 27.0–29.9 kg/m^2^ and at least one obesity-related comorbidity, while acknowledging their modest and time-limited efficacy, with typical total body weight loss of approximately 10–15% at 6 months and variable durability following device removal [8]. The guideline further emphasises that IGBs should be delivered within structured, multidisciplinary programmes incorporating dietary, behavioural, and medical support to optimise outcomes.

Similarly, the American Gastroenterological Association (AGA) clinical practice guideline on IGBs emphasises that IGBs should be used in conjunction with lifestyle modification and as part of broader weight-management strategies, rather than as standalone definitive treatments, often alongside pharmacotherapy and other interventions [15]. Both ASGE and ESGE recognise a potential role for IGBs as bridging therapy before bariatric surgery in higher-risk patients [16], but do not support their use as a long-term sole solution. Overall, contemporary guidelines position IGBs as minimally invasive, reversible interventions with a narrower, more selective role within multimodal obesity care.

Important safety considerations also inform current positioning. Early gastrointestinal intolerance is common, with nausea and vomiting frequently reported. Early removal is required in up to 10–20% of patients [17]. Although serious adverse events, including balloon migration, gastric perforation, and small-bowel obstruction, are uncommon, they reinforce the need for selective use of IGB therapy within a comprehensive, multidisciplinary weight-management pathway, consistent with contemporary ASGE–ESGE and AGA guidance [8,15]. Notably, endoscopic sleeve gastroplasty (ESG) represents an alternative endoscopic intervention with evidence of more sustained weight loss, further underscoring the time-limited role of IGBs [18,19].

## 4. Mechanisms of Action

The mechanisms underlying weight loss with IGBs are multifactorial. The primary effect is gastric volume occupation, which reduces functional gastric capacity and promotes early satiety [20]. By physically occupying space within the stomach, the balloon limits the volume available for food intake and thereby reduces caloric consumption per meal. In addition, several physiological studies have demonstrated delayed gastric emptying, contributing to prolonged postprandial fullness and correlating with reductions in total daily energy intake in controlled settings [21,22].

Neurohormonal mechanisms may also play a role. Ghrelin, an orexigenic hormone primarily secreted by gastric fundal cells, together with other appetite-regulating hormones, has been reported to change following balloon placement [23]. Several studies have shown suppression of ghrelin during IGB therapy, potentially contributing to reduced hunger signalling, although these effects are inconsistent and appear less pronounced than those observed after metabolic surgery or incretin-based pharmacotherapy [23,24]. Overall, the neuroendocrine adaptations to IGBs remain incompletely understood, with heterogeneous findings across study populations [24].

Behavioural reinforcement is another important component, as patients often adopt smaller portion sizes and improved dietary habits during the treatment period [25]. The physical restriction imposed by the balloon acts as a behavioural scaffold, prompting modification of eating behaviour out of physiological necessity rather than willpower alone. Repeated exposure to reduced portion sizes over the six-month treatment course may facilitate consolidation of new dietary habits, whereby the discomfort associated with overeating discourages excessive consumption, mirroring established principles of behavioural modification and habit formation [26].

However, unlike pharmacological therapies, which exert sustained central and peripheral effects, or surgical procedures that induce permanent anatomical changes, the effects of IGBs are inherently temporary, a limitation with important implications for long-term outcomes [27].

## 5. Clinical Effectiveness

The clinical effectiveness of IGBs is supported by systematic reviews and meta-analyses, including syntheses of randomised controlled trials, which demonstrate modest but clinically meaningful short-term weight loss compared with lifestyle-based or standard medical management [28,29,30,31,32]. Reported outcomes vary according to balloon type, baseline BMI, treatment duration, and the weight-loss metric used. Across these evidence syntheses, total body weight loss at 6 months is generally approximately 10–15%, while excess weight loss is commonly reported in the range of 25–40% [28,29,30,31].

A recent systematic review and meta-analysis of 15 randomised controlled trials (n = 1961) demonstrated significantly greater weight loss with IGBs compared with standard medical therapy, which includes dietary modification, physical activity, and pharmacological therapies. Pooled analyses showed higher percentage excess weight loss at 6 months (mean difference 16.80; 95% CI 9.22–24.38), 9 months (14.36; 95% CI 7.67–21.04), and 12 months (13.10; 95% CI 10.43–15.77), accompanied by greater reductions in total body weight and BMI. Outcomes varied according to balloon type and baseline BMI, underlining heterogeneity in treatment response and the importance of appropriate patient selection [32].

Evidence from systems designed for treatment beyond 6 months provides additional, albeit largely observational, context. In a prospective multicentre series of 1149 patients treated with the Orbera 365TM IGB (Apollo Endosurgery, Austin, TX, USA), weight loss continued until device removal at 52 weeks; among patients with weight recorded at removal, median TBWL was 15.38% and median EWL was 53.99%. Premature removal for intolerance occurred in 4.35% of patients [33]. A retrospective comparison of 6-month Orbera and 12-month Orbera 365 treatment found no significant difference in %TBWL at removal (15.3% versus 14.7%; *p* = 0.7) [34]. In contrast, a multicentre randomised trial of a 32-week adjustable IGB plus lifestyle intervention reported mean TBWL of 15.0%, compared with 3.3% with lifestyle intervention alone; upward balloon-volume adjustment provided an additional mean 5.2% TBWL, and weight loss was maintained for 6 months after removal [35].

Despite these outcomes, weight regain after balloon removal is common and represents a fundamental limitation of IGB therapy. A systematic review and meta-analysis reported that patients may regain approximately 35–50% of the weight lost within 6–12 months of removal in some settings [28]. Early removal due to intolerance has also been reported, with larger series describing premature removal in approximately 10–20% of patients because of persistent gastrointestinal symptoms [30]. The long-term outlook is similarly constrained: a 10-year follow-up of a double-blind randomised controlled trial (n = 99; 50 IGB versus 49 control) showed that, although IGB therapy achieved greater weight loss at 6 and 12 months, these differences were not sustained, and by 10 years there were no significant differences in weight, BMI, or obesity-related comorbidities between groups [36].

Patient-reported outcomes suggest high acceptability, with approximately 70–80% of patients reporting satisfaction at 6 months and improvements in appetite control and eating behaviour, even though early gastrointestinal adverse effects are common [37]. Early symptoms such as nausea, vomiting, and abdominal discomfort frequently occur and can lead to early discontinuation in a minority of patients [17]. While serious complications are rare, events including balloon migration, gastric perforation, and small-bowel obstruction have been described; case-based reports highlight small-bowel obstruction due to unrecognised balloon deflation as a delayed but potentially life-threatening event, with incidence rates reported below 0.2% [15,38]. These safety considerations reinforce the need for careful patient selection, close clinical monitoring, and structured follow-up within a multidisciplinary programme.

## 6. Psychological Aspects and IGBs as a Bridge to Bariatric Surgery

The psychological dimensions of IGB therapy represent an underappreciated but clinically relevant consideration. Obesity is frequently accompanied by psychological comorbidity, including depression, anxiety, and binge eating disorder, each of which may influence treatment engagement and long-term outcomes [39]. The treatment period can serve as a structured interval for behavioural adaptation, and patients may provide useful prognostic information regarding their suitability for bariatric surgery, where similar demands become permanent. In this way, IGBs may function as a psychological bridge, allowing multidisciplinary teams to identify and address maladaptive behaviours before proceeding to a definitive intervention [40].

IGBs may also serve as a practical bridge for patients unable to access or tolerate obesity pharmacotherapy. Contraindications to GLP-1 receptor agonists, intolerable adverse effects, or limited access related to cost and supply constraints may preclude their use in a meaningful proportion of patients, for whom IGBs can represent a viable alternative within multimodal care pathways [41,42].

Within this context, the role of IGBs appears increasingly selective rather than general. Their utility is greatest in specific patient subgroups: those with class I or II obesity who do not meet surgical thresholds yet have failed conservative measures; patients unable to use, tolerate, or access pharmacotherapy; individuals with severe obesity requiring preoperative weight reduction before bariatric surgery; those who are opposed to pharmacological or surgical intervention and prefer a time-limited, reversible endoscopic option; and patients managed within staged multimodal pathways, in whom IGB therapy may be used alongside dietary and behavioural support [8,15]. Limited comparative evidence suggests that pharmacotherapy may enhance weight loss or reduce post-removal weight regain [43]. However, the evidence is heterogeneous and prospective comparative studies are needed before a consistent additive benefit can be confirmed.

## 7. Comparison with Pharmacotherapy

In the evolving landscape of obesity management, IGBs must be considered alongside increasingly effective pharmacological therapies, particularly GLP-1 receptor agonists (GLP-1 RAs) [44]. While IGBs provide a minimally invasive, reversible intervention that achieves clinically meaningful short-term weight loss, their role is now interpreted in the context of the sustained efficacy and robust evidence base supporting modern pharmacotherapy. A direct comparison highlights key differences in mechanism, durability, clinical outcomes, adverse-event profiles, and positioning within current treatment pathways (Table 1).

IGBs generally achieve approximately 10–15% TBWL at 6 months. Meta-analytic estimates suggest a decline to approximately 10% at 12 months and approximately 7% at 18–24 months, although these longer-term data are limited, heterogeneous, and predominantly reflect outcomes after balloon removal [28,29,30,31]. Extended-duration balloon systems provide additional, largely observational evidence: 12-month Orbera 365 studies have reported approximately 15–16% TBWL at device removal [33,34,35]. In contrast, semaglutide and tirzepatide trials have reported weight loss of approximately 15% at 68 weeks and up to 20.9% at 72 weeks, respectively [10,11,12]. This difference in magnitude and durability of effect is central to understanding the evolving positioning of IGBs relative to pharmacotherapy.

In contrast, GLP-1 receptor agonists carry no procedural risk, with adverse effects predominantly limited to dose-dependent, transient gastrointestinal symptoms [61,62]. The absence of interventional risk with pharmacotherapy represents an advantage over device-based approaches, particularly in patients with elevated procedural risk [63]. Overall, safety considerations for IGBs highlight the need for careful patient selection, close clinical monitoring, and structured follow-up, especially given the device’s temporary nature and the broader shift towards long-term medical therapies in obesity care [64].

## 8. Health System and Cost Considerations

IGBs offer practical advantages, including outpatient delivery, reversibility, and relatively low procedural complexity, which may improve accessibility compared with surgery. However, their cost-effectiveness remains uncertain because of the limited durability of weight loss and the potential need for repeat interventions. A comprehensive economic evaluation of IGBs must account for the total cost of the procedural pathway rather than the device alone; this encompasses pre-procedure assessment, endoscopic placement under sedation, the device itself, dietetic and follow-up support, and a second endoscopic procedure for device retrieval at 6 or 12 months. Longer-duration balloons such as the Orbera 365, which require both endoscopic placement and removal, are associated with substantially higher procedure and programme costs than swallowable capsule balloons, reflecting device price, anaesthesia/endoscopy time, and follow-up requirements [65]. In the United Kingdom (UK), access to IGB therapy through publicly funded pathways appears limited, and provision may vary between local services [66]. The limited durability of weight loss following device removal further compounds cost-effectiveness concerns, as weight regain may necessitate repeat interventions, escalating cumulative expenditure [67].

Pharmacotherapy with GLP-1 RAs and dual GIP/GLP-1 agonists is similarly associated with substantial financial burden. In the UK, private access to semaglutide 2.4 mg (Wegovy) costs approximately £90–£305 per month depending on provider, with a reported mean price of approximately £209 for the 2.4 mg dose; initial consultation, prescription and follow-up arrangements may incur additional charges [68]. In England, NICE recommends semaglutide within a specialist weight-management service, for a maximum of two years, in adults meeting specified BMI and comorbidity criteria [69].

A balanced economic appraisal must therefore consider not only upfront costs but also durability of effect, the likelihood of retreatment, and systemic affordability. Health technology assessments, including NICE frameworks, increasingly prioritise interventions demonstrating sustained clinical benefit per unit cost. In this context, the superior durability and weight-loss magnitude of contemporary pharmacotherapy and bariatric surgery may favour their cost-effectiveness over repeated device-based interventions, aligning with health technology assessment priorities that emphasise durable clinical benefit and long-term value [70]. However, the outpatient, reversible nature of IGBs and their potential role in specific subgroups suggest that targeted use within specialist services may still be justified where resources permit [71].

## 9. Future Directions

The rapid evolution of obesity therapies emphasises the need to refine the positioning of IGBs within multimodal care pathways. Future research should prioritise direct comparative trials between IGB therapy and modern pharmacotherapies, particularly GLP-1 receptor agonists and dual GIP/GLP-1 agonists, as robust randomised data in this area remain limited, and optimal treatment protocols are yet to be defined. Such studies should evaluate not only weight loss and metabolic outcomes, but also durability, quality of life, patient preference, and real-world adherence [20,72].

The potential for synergistic effects when IGBs are integrated into multimodal protocols warrants dedicated investigation. Endoscopic interventions such as IGB placement may act as bridges or adjuncts to pharmacological or surgical treatments, particularly in patients with severe obesity, elevated procedural risk, or limited access to long-term pharmacotherapy. Carefully designed prospective trials and registry-based studies could explore staged algorithms in which IGBs are combined with dietary, behavioural, and pharmacological support, and assess whether such strategies enhance overall outcomes compared with monotherapy [20].

Finally, robust health economic analyses are needed to compare the cost-effectiveness of IGBs with contemporary pharmacotherapy, endoscopic sleeve gastroplasty, and bariatric surgery across different health systems. These should incorporate device and programme costs, retreatment rates, durability of effect, and impacts on obesity-related comorbidities [73]. As national bodies such as NICE increasingly prioritise interventions that deliver sustained benefit per unit cost, clarifying the economic profile of IGBs will be essential for rational resource allocation and guideline development [74].

## 10. Conclusions

IGBs provide modest but clinically meaningful short-term weight loss and can facilitate behavioural change, yet their impact is constrained by variable tolerability and limited long-term durability. In the context of highly effective pharmacotherapy and evolving endoscopic techniques, their role is increasingly selective rather than central.

Contemporary evidence and guideline recommendations support the use of IGBs as adjunctive or bridging therapies within structured, multimodal treatment frameworks rather than as primary intermediate options. Their greatest utility lies in carefully chosen patient subgroups, including those who do not meet surgical thresholds yet have failed conservative measures, those with contraindications or intolerance to pharmacotherapy, and individuals requiring preoperative weight reduction or preferring reversible endoscopic approaches.

The rapid emergence of highly effective GLP-1-based therapies challenge existing treatment algorithms and highlights the need to re-evaluate how device-based interventions such as IGBs are positioned within obesity care pathways. Ongoing research and updated guideline development will be essential to ensure that IGBs are used where they add genuine value and are integrated effectively into comprehensive, patient-centred obesity management.

## Figures and Tables

**Table 1:** Comparison of IGBs and GLP-1 RAs.

Domain	Intragastric Balloons (IGBs)	GLP-1 Receptor Agonists (GLP-1 RAs)
Mechanism	Gastric volume occupancy, delayed gastric emptying, neurohormonal effects [21,22]	Appetite suppression, delayed gastric emptying, central satiety signalling [45,46]
Delivery	Endoscopic placement; temporary (~6 months)	Subcutaneous injection; weekly or daily
Total body weight loss	~10–15% TBWL at 6 months [29,30,31]; longer-duration studies report ~15% TBWL at 12 months [33,34,35].	15% with semaglutide 2.4 mg, once weekly injection, at 68 weeks; 15.2% maintained at 104 weeks (STEP-1, STEP-5); up to 20.9% with tirzepatide 15 mg, once weekly injection, at 72 weeks (SURMOUNT-1) [10,11,12]
Durability	Limited; weight regain common after removal [28,47]	Sustained with continued therapy [12,46]
Evidence base	Moderate (randomised trials, meta-analyses; heterogeneity present) [29,30,31]	Strong (multiple large phase 3 randomised trials [RCT] in obesity: STEP-1, STEP-5, SURMOUNT-1) [10,11,12]
Cardiometabolic benefit	Modest improvements [22,48]	Modest to substantial improvements, depending on agent and duration [49,50,51]
Adverse effects	Nausea, vomiting, abdominal discomfort common; rare serious complications (e.g., migration, obstruction) [38]	Gastrointestinal symptoms (nausea, vomiting, diarrhoea, constipation) common; generally well tolerated; rare serious events such as pancreatitis [52,53,54]
Reversibility	Yes (device removal)	Yes (cessation of drug)
Role in guidelines	Adjunct or bridge therapy within structured programmes [8,15]	Central pillar of long-term medical management in recent obesity guidelines [55,56,57]
Cost-effectiveness	Uncertain due to limited durability and potential need for repeat interventions [28,58]	Increasing evidence of cost-effectiveness with sustained use and cardiometabolic benefit [59,60]

## Data Availability

No new data were created or analyzed in this study. Data sharing is not applicable.

## Abbreviations

The following abbreviations are used in this manuscript:

ASGE	American Society for Gastrointestinal Endoscopy
ASMBS	American Society for Metabolic and Bariatric Surgery
AGA	American Gastroenterological Association
BMI	Body mass index
EBT	Endoscopic Bariatric Therapy
ESGE	European Society of Gastrointestinal Endoscopy
ESG	Endoscopic sleeve gastroplasty
EWL	Excess weight loss
GIP	Glucose-dependent Insulinotropic Polypeptide
GLP-1	Glucagon-like peptide-1
GLP-1 Ras	Glucagon-like peptide-1 receptor agonists
IFSO	International Federation for the Surgery of Obesity and Metabolic Disorders
IGBs	Intragastric balloons
MBS	Metabolic and Bariatric Surgery
NHS	National Health Service
NICE	National Institute for Health and Care Excellence
RCT	Randomised controlled trial
TBWL	Total body weight loss
UK	United Kingdom
